# Phylogenetic analysis and antibiotic resistance of *Escherichia coli* isolated from wild and domestic animals at an agricultural land interface area of Salaphra wildlife sanctuary, Thailand

**DOI:** 10.14202/vetworld.2022.2800-2809

**Published:** 2022-12-08

**Authors:** Taksaon Duangurai, Amporn Rungruengkitkul, Thida Kong-Ngoen, Witawat Tunyong, Nathamon Kosoltanapiwat, Poom Adisakwattana, Muthita Vanaporn, Nitaya Indrawattana, Pornpan Pumirat

**Affiliations:** 1 Department of Companion Animal Clinical Sciences, Faculty of Veterinary Medicine, Kasetsart University, Bangkok, Thailand; 2 Department of Microbiology and Immunology, Faculty of Tropical Medicine, Mahidol University, Bangkok, Thailand; 3 Department of Helminthology, Faculty of Tropical Medicine, Mahidol University, Bangkok, Thailand

**Keywords:** antibiotic resistance, domestic animal, *Escherichia coli*, phylogenic groups, phylogeny, wild animal

## Abstract

**Background and Aim:**

Domestic and wild animals are important reservoirs for antibiotic-resistant bacteria. This study aimed to isolate *Escherichia coli* from feces of domestic and wild animals at an agricultural land interface area of Salaphra Wildlife Sanctuary, Thailand, and study the phylogenic characteristics and antibiotic resistance in these isolates.

**Materials and Methods:**

In this cross-sectional, descriptive study, we randomly collected ground feces from free-ranging wild animals (deer and elephants) and domestic animals (cattle and goats). All fecal samples were inoculated onto MacConkey agar plates, and lactose-fermenting colonies were identified as *E. coli*. Antibiotic susceptibility of the *E. coli* isolates was determined using the disc diffusion method. Polymerase chain reaction assays were used to detect antibiotic resistance and virulence genes.

**Results:**

We obtained 362 *E. coli* isolates from the collected fecal samples. The *E. coli* isolates were categorized into four phylogenetic groups according to the virulence genes (*chuA*, *vjaA*, and *TspE4C2*). Phylogenetic Group D was predominant in the deer (41.67%) and elephants (63.29%), whereas phylogenetic Group B1 was predominant in the cattle (62.31%), and phylogenetic Groups A (36.36%) and B2 (33.33%) were predominant in the goats. Antibiotic susceptibility testing revealed that most antibiotic-resistant *E. coli* were isolated from domestic goats (96.96%). Among the 362 *E. coli* isolates, 38 (10.5%) were resistant to at least one antibiotic, 21 (5.8%) were resistant to two antibiotics, and 6 (1.66%) were resistant to three or more antibiotics. Ampicillin (AMP) was the most common antibiotic (48.48%) to which the *E. coli* were resistant, followed by tetracycline (TET) (45.45%) and trimethoprim-sulfamethoxazole (3.03%). One isolate from an elephant was resistant to five antibiotics: AMP, amoxicillin, sulfisoxazole, TET, and ciprofloxacin. Determination of antibiotic resistance genes confirmed that *E. coli* isolates carried antibiotic resistance genes associated with phenotypic resistance to antibiotics. Most antibiotic-resistant *E. coli* belonged to phylogenic Groups A and B1, and most non-resistant *E. coli* belonged to phylogenic Groups B2 and D.

**Conclusion:**

Monitoring *E. coli* isolates from wild and domestic animals showed that all four phylogenic groups of *E. coli* have developed antibiotic resistance and are potential sources of multidrug resistance. High levels of antibiotic resistance have been linked to domestic animals. Our results support strengthening surveillance to monitor the emergence and effects of antibiotic-resistant microorganisms in animals.

## Introduction

Issues continue to emerge regarding antibiotic-resistant bacteria [1–4]. Infections with antibiotic-resistant bacteria significantly affect public health worldwide and present a global health problem [5, 6]. *Escherichia coli* is an important reservoir of antibiotic resistance [7–9].

*E. coli* is a part of the normal intestinal flora that colonizes mammalian gastrointestinal tracts [10] and is divided into eight phylogenetic groups (i.e., A, B1, B2, C, D, E, F, and clade I) based on the presence or absence of virulence genes [11, 12]. Commensal *E. coli* strains typically belong to groups A or B1. Most pathogenic strains responsible for intestinal infections belong to Groups A, B1, and D, whereas extraintestinal *E. coli* belong to groups B2 or D [13, 14]. These phylogenetic groups differ in terms of antibiotic resistance patterns, virulence genes, use of sugars, and environmental characteristics [15]. Although the role of *E. coli* in providing a reservoir for antibiotic resistance has been acknowledged [16, 17], the link between antibiotic resistance and phylogenetic characteristics of this bacterium remain uncertain. Moreover, limited data exist regarding *E. coli* in wild and domestic animals in Thailand.

Therefore, we aimed to examine *E. coli* from fecal samples of domestic goats and cattle fed on farmland surrounding the Salakpra Wildlife Sanctuary, Kanchanaburi Province, in Western Thailand, and from wild elephants and deer that live in this sanctuary to determine the phylogenetic groups and antibiotic resistance.

## Materials and Methods

### Ethical approval

The studied animals underwent no direct interventions associated with pain, suffering, or damage during the experiment. Fecal samples were collected from the ground for bacterial cultures. Therefore, these examinations required no announcement or permission with regard to the animal protection law because no experimental measures inflicted pain, suffering, or damage to these animals.

### Study period and location

The study was conducted from January to November 2013 at Salakpra Wildlife Sanctuary, Kanchanaburi Province, Thailand. Salakpra Wildlife Sanctuary is Thailand’s first wildlife sanctuary, located in the Western Forest Complex in Kanchanaburi Province. The area comprises mixed deciduous, dry dipterocarp, and dry evergreen forest and is home to Asian elephants (*Elephas maximus*), gaur *(Bos gauru*s), and sambar deer (*Rusa unicolo*r).

### Sample collection

In this cross-sectional, descriptive study, we collected 178 fecal samples from free-ranging wild and domestic animals around the Salakpra Wildlife Sanctuary. The 178 fecal samples were randomly collected from wild deer (n = 45), wild elephants (n = 55), domestic cattle (n = 62), and domestic goats (n = 16). Only fresh (moist) fecal samples were collected from the ground. A veterinary specialist identified the animal species by observing the fecal appearance and characteristics. The fecal material was sampled using sterile swabs, which were dipped in sterile Cary-Blair media (CM0519, Oxoid, Basingstoke, UK), kept in a cool box (0°C–4°C) and transferred to the laboratory at the Department of Microbiology and Immunology, Faculty of Tropical Medicine, Mahidol University, Bangkok, Thailand, within 24 h for *E. coli* identification.

### *Escherichia coli* isolation and identification

The fecal samples were inoculated onto MacConkey agar plates and incubated at 37°C for 24 h. Suspected *E. coli* colonies were examined through Gram staining and observed microscopically for bacterial morphology. Suspected *E. coli* colonies were identified by biochemical tests, including triple sugar iron agar, lysine decarboxylase, ornithine decarboxylase/deaminase, urea hydrolysis, indole production, and motility [18].

### Antibiotic susceptibility testing

*Escherichia coli* isolates were examined for resistance to the following 17 antibiotics using the Kirby-Bauer disk diffusion method according to Clinical Laboratory Standards Institute guidelines [19]: amoxicillin/clavulanic acid (AMC, 20 μg/10 μg), ampicillin (AMP, 10 μg), cephalexin (30 μg), cefoxitin (CEF, 30 μg), ceftiofur (30 μg), aztreonam (30 μg), imipenem (10 μg), meropenem (10 μg), ciprofloxacin (CIP, 5 μg), norfloxacin (10 μg), nalidixic acid (30 μg), chloramphenicol (CHL, 30 μg), trimethoprim-sulfamethoxazole (SXT, 1.25 μg/23.75 μg), gentamicin (GEN, 10 μg), tobramycin (TOB, 30 μg), amikacin (AMK, 30 μg), and tetracycline (TET, 15 μg) (Oxoid). *Escherichia coli* isolates resistant to at least one antibiotic in three or more classes were considered multidrug-resistant [20].

### DNA extraction and detection of antibiotic resistance genes

Total bacterial DNA was extracted from the *E. coli* isolates using a DNA extraction kit (Qiagen, Hilden, Germany) per the manufacturer’s instructions. Genomic DNA from the *E. coli* isolates was used as the DNA template to amplify the antibiotic resistance genes using polymerase chain reaction (PCR) with primers specific for individual genes (Table-1) [11, 12, 18]. The reaction was performed in a 25 µL mixture containing 2.5 µL 1X Taq buffer, 1 mM MgCl_2_, 0.2 mM dNTP, 1 µM each of the forward and reverse primers, and 2 units of *Taq* DNA polymerase (Thermo Fisher Scientific, USA). PCR was performed under the following conditions: denaturation for 5 min at 94°C; 30 cycles of 30 s at 94°C, 30 s at 60°C, and 30 s at 72°C; and a final extension step of 7 min at 72°C. Polymerase chain reaction amplicons were subjected to 1.5% agarose gel electrophoresis, stained with ethidium bromide, and visualized with an ultraviolet transilluminator.

**Table-1:** Polymerase chain reaction primers used in this study with expected product sizes for amplifying the *Escherichia coli* target genes.

Target genes	Primer sequence (5’- 3’)	Product size (bp)	Reference
Phylogenetic group			
*chuA*	GACGAACCAACGGTCAGGAT	279	[11]
	TGCCGCCAGTACCAAAGACA		
*yjaA*	CAAACGTGAAGTGTCAGGAG	211	[12]
	AATGCGTTCCTCAACCTGTG		
*TspE4.C2*	CACTATTCGTAAGGTCATCC	152	[12]
	AGTTTATCGCTGCGGGTCGC		
*arpA*	AACGCTATTCGCCAGCTTGC	400	[12]
	TCTCCCCATACCGTACGCTA		
Beta-lactams			
*bla* TEM	TTAACTGGCGAACTACTTAC	247	[18]
	GTCTATTTCGTTCATCCATA		
*bla* SHV	AGGATTGACTGCCTTTTTG	393	[18]
	ATTTGCTGATTTCGCTCG		
*bla* CMY-2	GACAGCCTCTTTCTCCACA	1,000	[18]
	TGGACACGAAGGCTACGTA		
Sulfonamide			
*sul1*	CGACACAGAAATCGAGCGTA	60	[18]
	GTCTTGCACCGAATGCATAA		
*sul2*	GGCAGATGTGATCGACCTCG	65	[18]
	ATGCCGGGATCAAGGACAAG		
*sul3*	GAGCAAGATTTTTGGAATCG	60	[18]
	AACTAACCTAGGGCTTTGGA		
Trimethoprim			
*dhfr1*	AAGAATGGAGTTATCGGGAATG	391	[18]
	GGGTAAAAACTGGCCTAAAATTG		
*dhfr*5	CTGCAAAAGCGAAAAACGG	432	[18]
	AGCAATAGTTAATGTTTGAGCTAAAG		
*dhfr*7	GGTAATGGCCCTGATATCCC	265	[18]
	TGTAGATTTGACCGCCACC		
Quinolone			
*qnrA*	AGAGGATTTCTCACGCCAGG	580	[18]
	TGCCAGGCACAGATCTTGAC		
*qnrB*	GGCATTGAAATTCGCCACTG	264	[18]
	TTTGCTGCTCGCCAGTCGAA		
*qnrS*	GCAAGTTCATTGAACAGGGT	428	[18]
	TCTAAACCGTCGAGTTCGGCG		
Aminoglycosides			
*aac* (3)-IIa (*aaa*C2)	CGGAAGGCAATAACGGAG	740	[18]
	TCGAACAGGTAGCACTGAG		
*aac* (3)-IV	GTGTGCTGCTGGTCCACAGC	627	[18]
	AGTTGACCCAGGGCTGTCGC		
*aad* B	GAGGAGTTGGACTATGGATT	208	[18]
	CTTCATCGGCATAGTAAAAG		
Tetracycline			
*tetA*	ATGATGGGTGCCTGTTTTCG	350	[18]
	CACCGACCATTACGCCA		
*tetB*	CGCCCAGTGCTGTTGTTGTC	173	[18]
	CGCGTTGAGAAGCTGAGGTG		
*tetC*	GCTGTAGGCATAGGCTTGGT	888	[18]
	GCCGGAAGCGAGAAGAATCA		

### Phylogenetic analysis

Phylogenetic determination was performed by amplifying the *chuA*, *vjaA*, *arpA*, and *TspE4.C2* genes [11, 12]. Multiplex PCR was performed using the recommended primers (Table-1). Each reaction was carried out and modified using a 20 µL mixture containing 10 µL 2X Green go Taq buffer enzymes, 20 pmoL of each primer, and 200 ng genomic DNA. Polymerase chain reaction was performed under the following conditions: denaturation for 5 min at 94°C; 30 cycles of 30 s at 94°C, 30 s at 55°C, and 30 s at 72°C; and a final extension step of 7 min at 72°C. The PCR products were resolved with 2% agarose gel electrophoresis, stained with ethidium bromide, and visualized with an ultraviolet transilluminator.

### Statistical analysis

All data were calculated using GraphPad Prism version 8 (La Jolla, CA, USA). The prevalence of phylogenetic group, antibiotic resistance phenotype, and genotype among *E. coli* isolates from feces of domestic and wild animals was presented as percentages.

## Results

### *Escherichia coli* isolation and identification

We collected 178 fecal samples from the ground from wild deer (n = 45), wild elephants (n = 55), domestic cattle (n = 62), and domestic goats (n = 16). of these samples, 136 were positive for E. coli and yielded 362 *E. coli* isolates. Of these 362 *E. coli* isolates,120, 79, 130, and 33 were isolated from 34 deer (75.56%; 34/45), 42 elephants (76.36%; 42/55), 48 cattle (77.42%; 48/62), and 12 domestic goats (75%; 12/16), respectively. Some samples yielded multiple isolates.

### Phylogenetic diversity

**Figure-1:**
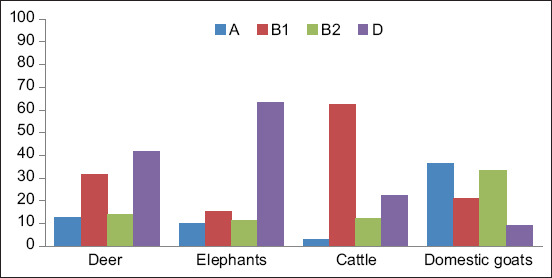
Prevalence of phylogenetic groups of Escherichia coli isolated from ground feces of wild and domestic animals.

The prevalence of the phylogenetic groups was determined based on *chuA*, *vjaA*, *arpA*, and *TspE4.C2* genes (Figure-1). Phylogeny detection revealed that phylogroup D was the predominant phylogenetic group from deer (41.67%) and elephants (63.29%), whereas phylogroup B1 was predominant in cattle (62.31%). The *E. coli* isolated from domestic goats were mainly from phylogroups A and B2 (36.36% and 33.33%, respectively).

### Antibiotic-resistant phenotypes

**Figure-2:**
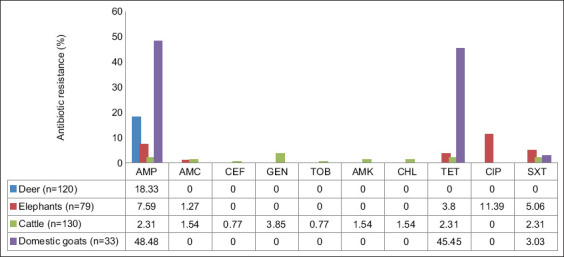
Percentage (%) of antibiotic resistant *Escherichia coli* isolated from ground feces of wild and domestic animals. AMP=Ampicillin, AMC=Amoxicillin/clavulanic acid, CEF=Cefoxitin, GEN=Gentamicin, TOB=Tobramycin, AMK=Amikacin, CHL=Chloramphenicol, TET=Tetracycline, CIP=Ciprofloxacin, SXT=Trimethoprim-sulfamethoxazole.

Figure-2 shows the antibiotic resistance test results. Of the *E. coli* isolates, 362 were resistant to 10 of 17 antibiotics. *Escherichia coli* isolated from deer showed only AMP resistance (18.33%). *Escherichia coli* isolates from elephants showed resistance to AMP (7.59%), amoxicillin/clavulanate (1.27%), TET (3.8%), CIP (11.39%), and SXT (5.06%). *Escherichia coli* isolates from cattle showed resistance to AMP (32.31%), amoxicillin/clavulanate (1.54%), CEF (0.77%), GEN (3.85%), TOB (0.77%), AMK (1.54%), CHL (1.54%), TET (2.31%), and SXT (2.31%). *Escherichia coli* isolates from domestic goats showed resistance to AMP (48.48%), TET (45.45%), and SXT (3.03%). All animal species tested contained AMP-resistant *E. coli* in their feces. The *E. coli* isolates from cattle showed resistance to most antibiotics (Figure-2). Conversely, the *E. coli* isolates from deer showed resistance to only one antibiotic (Figure-2).

Three animal species in our study contained multidrug-resistant *E. coli*: cattle, domestic goats, and elephants. Of the *E. coli* isolates from elephants, 1.27%, 3.8%, 1.27%, and 1.27% showed multidrug-resistant phenotypes to two, three, four, and five antibiotics, respectively. Of the *E. coli* isolates from cattle, 1.5%, 2.31%, and 0.77% were resistant to two, three, and four antibiotics, respectively. Of the *E. coli* isolates from domestic goats, 6.06%, 33.33%, and 3.03% were multidrug-resistant to two, three, and four antibiotics, respectively. Twenty multidrug-resistance patterns were identified from all fecal isolates (Table-2).

**Table-2:** Antibiotic resistance patterns in *Escherichia coli* isolated from feces of deer, elephants, cattle, and domestic goats.

Antibiotic-resistant patterns	Number of isolates	Source of feces
AMP	25	Deer, goats
AMP + SXT	3	Elephants, cattle
AMP + AMK	1	Cattle
AMK	1	Cattle
AMP + TET	12	Goats
AMP + TET + CIP	1	Elephants
AMP + SXT + TET + GEN	1	Cattle
AMP + AMC + SXT + TET+ CIP	1	Elephants
AMP + SXT + TET	1	Goats
AMP + AMC + TET	1	Goats
TET	2	Cattle, goats
AMC	2	Cattle
AMP + CIP	2	Elephants
CIP + TET	1	Elephants
CIP	4	Elephants
SXT	1	Elephants
GEN	3	Cattle
GEN + CHL	1	Cattle
SXT + TET	1	Cattle
CEF + TOB + CHL	1	Cattle

AMP=Ampicillin, AMC=Amoxicillin/clavulanic acid, CEF=Cefoxitin, GEN=Gentamicin, TOB=Tobramycin, AMK=Amikacin, CHL=Chloramphenicol, TET=Tetracycline, CIP=Ciprofloxacin, SXT=Trimethoprim-sulfamethoxazole

Each phylogenetic group of *E. coli* showed different resistances to the tested antibiotics (Table-3). *Escherichia coli* phylogenetic Group A exhibited resistance to AMP, AMC, GEN, TET, CIP, and SXT. Group B1 showed resistance to AMP, CEF, GEN, AMK, CHL, TET, CIP, and SXT. Group B2 showed resistance to AMP, AMC, TET, CIP, and SXT. Group D exhibited resistance to AMP, AMC, GEN, TET, CIP, and SXT.

**Table-3:** Phylogenetic *Escherichia coli* groups among animal species resistant to antibiotics.

Antibiotic resistance pattern	Phylogenetic Group			

	A	B1	B2	D
Ampicillin-resistant isolates				
Deer	5	35	12	23
Elephants	1	1	0	2
Cattle	3	1	0	0
Domestic goats	10	2	2	0
Amoxicillin clavulanate-resistance isolates				
Deer	0	0	0	0
Elephants	1	0	0	0
Cattle	0	0	1	1
Domestic goats	0	0	0	0
Cefoxitin-resistance isolates				
Deer	0	0	0	0
Elephants	0	0	0	0
Cattle	0	1	0	0
Domestic goats	0	0	0	0
Gentamicin-resistance isolates				
Deer	0	0	0	0
Elephants	0	0	0	0
Cattle	1	2	0	2
Domestic goats	0	0	0	0
Tobramycin-resistance isolates				
Deer	0	0	0	0
Elephants	0	0	0	0
Cattle	0	1	0	0
Domestic goats	0	0	0	0
Amikacin-resistance isolates				
Deer	0	0	0	0
Elephants	0	0	0	0
Cattle	0	2	0	0
Domestic goats	0	0	0	0
Chloramphenicol-resistance isolates				
Deer	0	0	0	0
Elephants	0	0	0	0
Cattle	0	2	0	0
Domestic goats	0	0	0	0
Tetracycline-resistance isolates				
Deer	0	0	0	0
Elephants	1	0	1	1
Cattle	2	1	0	0
Domestic goats	8	3	3	0
Ciprofloxacin-resistance isolates				
Deer	0	0	0	0
Elephants	1	1	1	5
Cattle	0	0	0	0
Domestic goats	0	0	0	0
Trimethoprim-sulfamethoxazole - resistance isolates				
Deer	0	0	0	0
Elephants	1	0	1	2
Cattle	3	0	0	0
Domestic goats	0	1	0	0

We also compared the *E. coli* phylogenetic groups among animal species (Table-3). Most *E. coli* isolates were resistant to AMP (66.44%, 97/146; Table-3); this AMP resistance occurred in all phylogenetic groups and all animal species studied. Ampicillin-resistant *E. coli* from deer were mostly in Group B1 (46.67%, 35/75), whereas those from domestic goats were mainly in Group A (71.43%, 10/14). Group B1 showed the highest prevalence of antibiotic resistance (36.3% and 53/146). Table-3 shows the low prevalence of resistance to other antibiotics and the determined phylogenetic groups.

### Prevalence of antibiotic resistance genes

*bla*TEM, *bla*SHV, *bla*CMY-2, *aaa*C2, *aac*(3)-IV, *aad*B, *tet*A, *tet*B, *tet*C, *qnr*A, *qnr*B, *qnr*S, *sul1*, *sul2*, *sul3*, *dfra1*, *dfra5*, and *dfra7* were identified from the 362 *E. coli* isolates in our study (Table-4). The total resistance-gene frequencies were *bla*TEM: 24.24%; *bla*SHV: 21.21%; *bla*CMY-2: 31.31%; *sul1:* 0%; *sul2:* 0%; *sul3:* 2.02%; *DfrA1:* 1.01%; *DfrA5:* 1.01%; *DfrA7:* 2.02%; *qnrA*: 1.01%; *qnrB*: 0%; *qnrS*: 0%; *aac*(3)-IIa: 0%; *aac*(3)-IV: 2.02%; *aadA*: 2.02%; *aadB*: 0%; *tetA*: 5.05%; *tetB*: 3.03%; and *tetC*: 0%. Fascinatingly, *bla*TEM*, bla*SHV*_,_* and *bla*CMY-2 were identified in *E. coli* isolates from all animal species and were phenotypically resistant to AMP. *aac*(*3*)IV and *aadA* were identified in *E. coli* isolates from cattle, which were phenotypically resistant to GEN. *tetA* and *tetB* were identified in *E. coli* isolates from elephants, cattle, and domestic goats. These results are consistent with our results that *E. coli* isolates from elephants, cattle, and domestic goats were resistant to TET. *qnrA* was identified in *E. coli* isolates from elephants. The data showed that only *E. coli* isolates from elephants were resistant to CIP. *Escherichia coli* isolates from elephants, cattle, and domestic goats that contained *sul3*, *dfra1*, *dfra5*, and *dfra7* were resistant to SXT. *sul3* is associated with sulfonamide resistance in *E. coli*. In addition, the presence of *dfra* is linked to trimethoprim resistance in *E. coli*.

**Table-4:** Occurrence of antibiotic resistance genes among antibiotic-resistant *Escherichia coli* isolates among animal species.

Antibiotic resistance genes	Gene occurrence (%)			

	Deer	Elephants	Cattle	Domestic goats
β-lactams				
*bla*TEM	25.33 (19/75)	80 (4/5)	20 (1/5)	-
*bla*SHV	25.33 (19/75)	1.27 (1/5)	20 (1/5)	-
*bla*CMY-2	26.67 (20/75)	-	40 (2/5)	64.29 (9/14)
Total	77.33 (58/75)	100 (5/5)	80 (4/5)	64.29 (9/14)
Aminoglycosides				
*aac (3) IIa*	-	-	-	-
*aac* *(3*) *IV*	-	-	25 (2/8)	-
*aadA*	-	-	25 (2/8)	-
*aadB*	-	-	-	-
Total	-	-	50 (4/8)	-
Tetracycline				
*tetA*	-	66.67 (2/3)	66.67 (2/3)	7.14 (1/14)
*tetB*	-	-	33.33 (1/3)	14.29 (2/14)
*tetC*	-	-	-	-
Total	-	66.67 (2/3)	100 (3/3)	21.43 (3/14)
Fluoroquinolone				
*qnrA*	-	12.5 (1/8)	-	-
*qnrB*	-	-	-	-
*qnrS*	-	-	-	-
Total	-	12.5 (1/8)	-	-
Trimethoprim-sulfamethoxazole				
*sul1*	-	-	-	-
*sul2*	-	-	-	-
*sul3*	-	25 (1/4)	33.33 (1/3)	-
*dfra1*	-	-	33.33 (1/3)	-
*dfra5*	-	25 (1/4)	-	-
*dfra7*	-	-	33.33 (1/3)	100 (1/1)
Total	-	50 (2/4)	100 (3/3)	100 (1/1)

## Discussion

In this study, we aimed to examine the antibiotic sensitivity pattern and phylogenetic variation of *E. coli* isolates from various wild and domestic animals in forests, residences, and agricultural interfaces in Thailand. Antibiotics are used to treat and prevent bacterial infections in people and animals [21]. However, antibiotic resistance has become highly prevalent among bacterial isolates worldwide [1–4]. It renders a serious problem for antibiotic therapy [5, 6]. Therefore, surveillance of antibiotic-resistant bacteria is necessary to control antibiotic use. *Escherichia coli* is an important reservoir for tracking bacterial antibiotic resistance because *E. coli* has the ability to transfer genetic material to and from other bacterial species and can harbor several resistance determinants [17]. Consequently, antibiotic resistance surveillance programs often use *E. coli* as an indicator [7–9]. The prevalence of antibiotic-resistant *E. coli* is increasing in many animals, such as poultry [22, 23], swine [24–26], cattle [18, 27, 28], and wild animals [29–32]. At present, limited data exist on antibiotic-resistant *E. coli* and their phylogenetic groups in wild and domestic animals in Thailand.

In Thailand, antibiotics are used on livestock farms to treat sick animals and support healthy animals during stressful periods to prevent bacterial infections. However, these bacteria can develop resistance to antibiotics. In addition, antibiotic-resistant bacteria can be transferred to other bacterial species in animals that might never be treated with antibiotics. Nowadays, one of the biggest obstacles to controlling and treating infectious diseases in these animals is the resistance of pathogenic bacteria to antibiotics. Thus, monitoring antibiotic-resistant *E. coli* in domestic and wild animals highlights the problem of antibiotic resistance among animals in Thailand. Here, we studied fecal samples from wild animals at Salakpra Wildlife Sanctuary in Kanchanaburi Province, the first wildlife sanctuary in Thailand. The diversity of wildlife in Salakpra is vast, with 352 animal species, including wild elephants and deer. Small-scale livestock farms also harbor domestic animals on the boundary of Salakpra. Domestic animal feces in this study were derived from cattle and goats.

We analyzed 362 *E. coli* isolates from 178 fecal samples from free-ranging herbivores, including wild elephants and deer and domestic cattle and goats. Phylogenetic analysis of the virulence genes *chuA*, *vjaA*, *arpA*, and *TspE4.C2* yielded four *E. coli* phylogenetic groups: A, B1, B2, and D. In deer and elephants, most *E. coli* were in phylogroup D. For domestic goats, most *E. coli* isolates were phylogenetic Groups A and B2. In *E. coli* from cattle, Group B1 was the predominant phylogenetic group. Consistent with the previous finding, Groups B2 and A were found mostly in *E. coli* isolated from domestic goats [33, 34]. *Escherichia coli* isolated from herbivorous wildlife in another study (auroch, buffalo, and waterbuck) were mostly in group B1 [35], which is consistent with our findings for cattle. Conversely, a previous study reported that *E. coli* strains from wild elephants belonged mostly to phylogenetic group B1 [36]. One study reported that group D *E. coli* are more likely to be pathogenic and are associated with pigs and humans [15]. However, group D *E. coli* isolated in our study did not adhere to or invade Caco2 cells (data not shown), suggesting that they are commensal and do not cause disease. In addition, most *E. coli* phylogenetic groups in the environment tend to belong to Group B1 [37]. The *E*. *coli* isolates in our study were clustered into different phylogenetic groups, indicating minimal contamination from the ground environment.

We analyzed the diversity of the *E. coli* phylogenetic groups and antibiotic resistance among wild and domestic animals. Phylogenetic Group B1 was the largest *E. coli* group with antibiotic resistance in wild and domestic animals. This is similar to a previous study on calves and chicken carcasses, which showed that *E. coli* Group B1 had the highest antibiotic resistance [38]. Conversely, one study found that *E. coli* Groups B2 and D commonly resist many antibiotics in domestic animals [39]. We found that different *E. coli* phylogenetic groups from different animals were resistant to the same antibiotics. Ampicillin-resistant *E. coli* from deer belonged mostly to phylogenetic Group B1, whereas AMP-resistant *E. coli* from domestic goats belonged mostly to phylogenetic group A. Thus, distributions of *E. coli* phylogenetic groups may influence the antibiotic resistance profiles. Additional research is required to explore the shared attributes in phylogenies that allow antibiotic resistance.

The *E. coli* isolated from wild and domestic animals in the area around the Salakphra Wildlife Sanctuary were resistant to ten antibiotics. Antibiotic resistance in commensal *E. coli* has been reported in various wild mammals worldwide, including birds [40–43], rodents [41], ungulates [44], primates [45, 46], boars [47], foxes [48], rabbits [48, 49], and deer [48, 50]. We found that *E. coli* isolates from deer were resistant to AMP. Other researchers have found that *E. coli* isolates from deer are resistant to other antibiotics. Tamamura-Andoh *et al*. [50] reported that *E. coli* isolates from deer were resistant to TET. Smith *et al*. [43] found that *E. coli* isolates from deer were resistant to oxacillin, penicillin, TET, and rifampin. Alonso *et al*. [51] found that *E. coli* isolates from deer were resistant to TET and quinolones. Li *et al*. [52] reported that *E. coli* isolates from deer were resistant to sulfonamides, streptomycin, and TET. Sarker *et al*. [53] found antibiotic resistance to AMP and sulfamethoxazole, TET, nalidixic acid, erythromycin, and CHL in *E. coli* isolates from deer in safari parks in Bangladesh. Conversely, Wasyl *et al*. [54] found no antibiotic resistance in *E. coli* isolates from wild deer in Poland.

In our study, *E. coli* isolates from wild elephants were resistant to five antibiotics: AMP, amoxicillin/clavulanate, TET, CIP, and SXT. This finding is similar to a report by Jayasekara *et al*. [55], who found that *E. coli* isolates from wild elephants were resistant to AMP, amoxicillin/clavulanate, TET, nitrofurantoin, and cefuroxime. However, CIP-resistant *E. coli* were isolated only from wild elephants in our study. Further investigation is needed to determine the environmental source of the antibiotic-resistant *E. coli* that spread to these wild elephants.

We found that *E. coli* isolates from cattle were resistant to several antibiotics, including AMP, amoxicillin/clavulanate, CEF, GEN, TOB, CHL, TET, and SXT. This is consistent with previous studies that found that *E. coli* from beef cattle were resistant to CEF and SXT [56]. These antibiotics are commonly used in cattle for routine treatments. Moreover, another report demonstrated that *E. coli* isolates from cattle exhibited resistance to AMP, ceftiofur, CHL, florfenicol, spectinomycin, and TET [57]. One study reported that *E. coli* isolated from cattle were resistant to aminoglycosides and TET [58]. *Escherichia coli* isolated from cattle can also resist TET [27]. These findings suggest a problematic increase in antibiotic-resistant *E. coli* in domestic cattle.

Both cattle and domestic goats are popular among livestock smallholders in Thai rural areas. Our study highlights that antibiotic resistance problems in domestic goats in Thailand should be addressed. *Escherichia coli* isolates from domestic goats were resistant to three antibiotics: AMP, TET, and SXT. In domestic goats, AMP showed the highest percentage of antibiotic resistance. Consistent with a previous report from Bangladesh, high levels of resistant *E. coli* isolates from goats were found against AMP and AMC, followed by SXT, TET, streptomycin, and GEN [59]. In Kenya, *E. coli* isolates from goats showed more frequent resistance to AMP, AMC, TET, ceftriaxone, and CHL [60]. In Switzerland, most isolates from goats were resistant to TET, streptomycin, and AMP [61]. In Michigan, USA, *E. coli* isolates from wild and domestic animals were most frequently resistant to TET, cephalothin, sulfisoxazole, and streptomycin [62]. These findings highlight the problem of antibiotic resistance among livestock in Thailand and other countries.

Our results showed multidrug resistance among *E. coli* isolates from elephants, cattle, and domestic goats, indicating the emergence of multidrug resistance among *E. coli* in wild and domestic animals in Thailand. One possible reason for the multidrug-resistant bacteria in wild animals is increased cohabitation between wild and domestic animals because deforestation causes wild animals to migrate into agricultural landscapes to survive. This allows the transmission of bacterial antibiotic resistance between wild and domestic animals [63]. Occurrences of multidrug-resistant *E. coli* have been reported in wildlife and domestic mammals elsewhere [64], for example, among sympatric wildlife, humans, livestock, and their shared environment in Kenya [65].

Antibiotic resistance among *E. coli* isolates in this study was confirmed by determining their antibiotic resistance genes, including *bla*TEM, *bla*SHV, *bla*CMY-2, *aac*C2, *aac*(*3*)*IV*, *aadB*, *tetA*, *tetB*, *tetC*, *qnrA*, *qnrB*, *qnrS*, *sul1*, *sul2*, *sul3*, *dfra1*, *dfra5*, and *dfra7*. Our *E. coli* isolates carried antibiotic resistance genes associated with phenotypic resistance to antibiotics. Most of these isolates from both wild and domestic animals contained antibiotic resistance genes to b-lactams. Thus, *E. coli* may transfer antibiotic resistance genes to other bacteria in these animals under certain conditions.

Although *E. coli* isolates in this study were diverse in terms of phylogenetic groups and antibiotic resistance, *E. coli* from both wild and domestic animals showed resistance to AMP. *Escherichia coli* isolates from elephants were resistant to TET and SXT, which was similar to the *E. coli* isolates from cattle and domestic goats, indicating that transfer of antibiotic-resistant *E. coli* may occur between wild and domestic animals. One possible explanation is that bacterial resistance may have spread in the study area through the dissemination of antibiotic resistance among bacterial populations in domestic and wild animals. In addition, Guenther *et al*. [64] suggested that antibiotic resistance may develop in the gut flora of wild mammals when the animals consume antibiotics in feed and water from nearby farms and indirectly after exposure to farm waste.

## Conclusion

The emergence and spread of antibiotic-resistant bacteria in natural environments are a major concern with serious implications for human and animal health. In this study, the examination of *E. coli* isolates from the ground feces of domestic and wild animals highlights the problem of antibiotic resistance. The prevalence of antibiotic-resistant *E. coli* may be due to the overuse of antibiotics in veterinary medicine or non-methodical use in feeding domestic animals. Furthermore, domestic animals may be reservoirs for spreading resistant *E. coli* to wild animals. Our findings intended to support the awareness of responsible use of antibiotics in animals. However, our investigation is restricted to speculation on the potential role of *E. coli* as a reservoir for antibiotic resistance in domestic and wild animals. More investigations are warranted. Probably, antibiotic resistance of a panel of gut microbiota is more rational and worthwhile. In addition, molecular insight of antibiotic-resistant mechanisms of bacteria is needed to clarify future development in the prevention and control of antibiotic resistance, as well as the treatment of the diseases.

## Authors’ Contributions

TD, NI, and PP: Conceived and designed the study. TD, AR, TK, WT, NK, PA, MV, NI, and PP: Performed the experiments and analyzed the data. TD and PP: Performed literature search and drafted the manuscript. NI and PP: Reviewed and edited the manuscript. TD and PP: Performed final manuscript revision. All authors have read and approved the final manuscript.
